# Supplementary material for: Are political-opinion pollsters missing ambivalence: “I love Trump”… “I hate Trump”
Source: PLoS One. 2021 Mar 11;16(3):e0247580. doi: 10.1371/journal.pone.0247580 (PMC7951855; doi:10.1371/journal.pone.0247580)
Supplement: Attachment — Submitted filename: Response to PLOS ONE Reviewers - January 2021.docx [file pone.0247580.s002.docx]

28 January 2021

James Camparo, Ph.D.

Physical Sciences Laboratories

The Aerospace Corporation

Dr. Dan Braha

Academic Editor,

PLOS ONE

Dear Dr. Braha

We wish to thank you and the reviewer for your careful reading of our manuscript, and the informative and useful comments. Below, we respond to those comments.

Academic Editor

1. The Academic Editor had one comment, which really asked us to consider the possibility of a relationship between ambivalence and voting contagion.

In reviewing the literature on contagion, we can see several ways in which contagion could actually be a latent factor related to ambivalence. We address this in lines 526 through 538, where we write

*Another potential latent factor could be ambivalence arising from contagion: the influence of social networks on attitudes (see for example [29] and references therein). For example, some individuals could be more susceptible/vulnerable to others’ opinions or views, perhaps due to age, personality, or living conditions (i.e., living alone as opposed to living with a partner). Continuing with this speculation, it may be that ambivalent individuals have social networks with close acting peer-nodes comprising both Democrats and Republicans. For example, an individual living in a family with some members identifying as Democrats and others as Republicans may be more likely to develop an ambivalent attitude towards President Trump. Alternatively, it could be that individuals with generable ambivalence tend to form peer networks with others of similar potential. The development of ambivalence could then be a “feedback loop” process, with these individuals stimulating the formation of each other’s ambivalent attitudes.*

Reviewer #1

1. This reviewer had three concerns. The first dealt with whether or not our questionnaire items were generating ambivalence, or instead a bimodal response arising from too generally phrased questionnaire items.

In response to this concern, it’s important to note that the items were not generated by us, but were taken from actual Quinnipiac and Brookings Institute surveys. Our purpose was to examine whether professionally developed surveys might be missing important information. In responding ambivalently (or bimodally due to too generally phrased an item), we show that professional survey items are apparently missing information.

We also argue, however, that there is really no distinction between an ambivalent response to a questionnaire item and a bimodal response due to too general a questionnaire item. As we now write on lines 507 through 525

*One way that latent factors could manifest in ambivalence is if questionnaire items lend themselves to different interpretations. For example, in Study 2 Item 25 states “I think the nation’s economy is getting better.” On the one hand, a participant could consider this item in the context of the stock market and think to answer Agree; on the other hand, this same participant could consider this item in the context of America’s widening wealth-gap and think to answer Disagree. It should be recognized, however, that this interpretive conflict fits the definition of ambivalence: “the simultaneous occurrence of positive and negative implicit or explicit evaluations of a single attitude object” [10], where the attitude object here is the economy taken broadly. In this situation the identification of ambivalence can alert the pollster or researcher to probe more deeply for latent factors (e.g., stock market and wealth-gap). To the extent that the pollster can identify well-delineated latent factors through follow-up probing, more detailed information has been gained on the participant’s evaluation of the attitude object. Of course, latent factors resolving ambivalence will rarely be so obvious (if present at all). More often we expect the presence of latent factors to force a fundamental re-appraisal of the attitude object (e.g., the political issue targeted by the questionnaire item). For example, one could easily imagine that latent factors drive ambivalence towards affirmative action, though teasing those latent factors apart could be a Gordian Knot puzzle.*

2. The Reviewer’s second concern was our making a distinction between party and ideology. In particular the Reviewer suggested that we focus “on either party… or ideology, it would make your paper more concise and cogent.”

Though we appreciate the Reviewer’s comment and suggestion, we disagree. While party and ideology will be correlated, there is strong empirical evidence suggesting that these are two independent constructs. We provide a citation validating this point, as we explain in lines 274 through 282:

*Though political ideology is certainly linked to party affiliation, Barber and Pope [25] have shown that party identification can be much stronger than ideology in terms of attitude expression. Further, during the 2020 presidential election it became clear that Democrats as a party were composed of ideologically distinct moderates and progressives, whereas Republicans as a party were composed of ideologically distinct ardent Trump supporters and traditional conservatives. Given these empirical results, we felt it important to explore potential ambivalence differences related to political ideology in addition to party affiliation.*

3. The Reviewer’s third concern related to the fact that it is well known that Independents have leanings (either Republican or Democrat), and that “[I]t is probably worth mentioning this and citing the literature.” We now write in lines 486 through 493

*To be clear, the ambivalence uncovered here is not ambivalence of Independents as a group. It is well known that roughly 46% of Independents lean Democratic, 35% of Independents lean Republican, and that only 19% have no leaning [28]. Thus, as a group Independents likely have ambivalence towards Donald Trump’s presidency. However, the ambivalence reported here is at the individual level. Thus, our results suggest that those Independents leaning Democratic or leaning Republican show greater levels of ambivalence at the individual level than those individuals who declare themselves Democrats or Republicans.*

Again, we wish to thank you and the reviewer for your reading of our manuscript, and your comments.

Sincerely,

Dr. James Camparo
